# Supplementary material for: A Comprehensive Assessment of Ultraviolet-Radiation-Induced Mutations in Flammulina filiformis Using Whole-Genome Resequencing
Source: J Fungi (Basel). 2024 Mar 20;10(3):228. doi: 10.3390/jof10030228 (PMC10971301; doi:10.3390/jof10030228)
Supplement: Supplementary file 1 [file jof-10-00228-s001.zip › Supplementary Material S8/KEGG annotation/out/64381550635650.os/KO/out_map/map03040.html]

KEGG PATHWAY: Spliceosome - Reference pathway


|  |  |
| --- | --- |
| **Spliceosome - Reference pathway** |  |

[
Pathway menu
| Organism menu
| Pathway entry
| Show description
| User data mapping
]

After transcription, eukaryotic mRNA precursors contain protein-coding exons and noncoding introns. In the following splicing, introns are excised and exons are joined by a macromolecular complex, the spliceosome. The standard spliceosome is made up of five small nuclear ribonucleoproteins (snRNPs), U1, U2, U4, U5, and U6 snRNPs, and several spliceosome-associated proteins (SAPs). Spliceosomes are not a simple stable complex, but a dynamic family of particles that assemble on the mRNA precursor and help fold it into a conformation that allows transesterification to proceed. Various spliceosome forms (e.g. A-, B- and C-complexes) have been identified.

|  |  |
| --- | --- |
| Reference pathway | 100% |
